# Fungicides and the Grapevine Wood Mycobiome: A Case Study on Tracheomycotic Ascomycete *Phaeomoniella chlamydospora* Reveals Potential for Two Novel Control Strategies

**DOI:** 10.3389/fpls.2019.01405

**Published:** 2019-10-31

**Authors:** Giovanni Del Frari, Alex Gobbi, Marie Rønne Aggerbeck, Helena Oliveira, Lars Hestbjerg Hansen, Ricardo Boavida Ferreira

**Affiliations:** ^1^LEAF—Linking Landscape Environment Agriculture and Food, Instituto Superior de Agronomia, University of Lisbon, Lisbon, Portugal; ^2^Department of Environmental Science, Section for Environmental Microbiology and Biotechnology, Environmental Microbial Genomics Group (EMG), Aarhus University, Roskilde, Denmark

**Keywords:** fungicides, *Phaeomoniella chlamydospora*, grapevine trunk diseases, mycobiome, microbial ecology

## Abstract

*Phaeomoniella chlamydospora* is a tracheomycotic fungus that colonizes the xylem of grapevines (*Vitis vinifera* L.), causing wood discoloration, brown wood streaking, gummosis, and wood necrosis, which negatively affect the overall health, productivity, and life span of vines. Current control strategies to prevent or cope with *P. chlamydospora* infections are frequently ineffective. Moreover, it is unclear how fungicides commonly applied in vineyards against downy and powdery mildew agents affect the wood mycobiome, including wood pathogens such as *P. chlamydospora*. In this study, we used next-generation sequencing to assess the effects of foliar spray of grapevines with inorganic (copper oxychloride and sulfur), synthetic (penconazole and fosetyl-aluminum), and natural (Blad) fungicides currently used against the downy and powdery mildews. The subjects of our investigation were (i) the resident wood mycobiome, (ii) the early colonization by a consortium of fungal wood endophytes (ACEA1), (iii) the wood colonization success of *P. chlamydospora*, and (iv) the *in planta* interaction between *P. chlamydospora* and ACEA1, under greenhouse conditions, in rooted grapevine cuttings of cv. Cabernet Sauvignon. The data obtained suggest that the resident mycobiome is affected by different fungicide treatments. In addition, the early colonization success of the endophytes composing ACEA1 varied in response to fungicides, with relative abundances of some taxa being overrepresented or underrepresented when compared with the control. The wood colonization by *P. chlamydospora* comported significant changes in the mycobiome composition, and in addition, it was greatly affected by the foliar spray with Blad, which decreased the relative abundance of this pathogen 12-fold (4.9%) when compared with the control (60.7%) and other treatments. The presence of the pathogen also decreased considerably when co-inoculated into the plant with ACEA1, reaching relative abundances between 13.9% and 2.0%, depending on the fungicide treatment applied. This study shows that fungicides sprayed to prevent infections of powdery and downy mildews have an effect on non-target fungi that colonize the endosphere of grapevines. We suggest two potential control strategies to fight *P. chlamydospora*, namely, the foliar spray with Blad and the use of ACEA1. Further studies to confirm these results are required.

## Introduction

Protecting grapevines (*Vitis vinifera* L.) from fungal pests by means of fungicides dates back to the late 1800s, with the accidental discovery that a mixture of copper sulfate and lime, sprayed on vine leaves, was not only able to deter thieves from stealing grapes but also able to prevent downy mildew infections (Morton and Staub, 2008). Since then, research on fungicides improved the use of copper and sulfur as contact fungicides and introduced additional synthetic broad-spectrum systemic fungicides (e.g., benzimidazoles, triazoles, fludioxonil, and organophosphorus compounds) during the mid-late 1900s (Morton and Staub, 2008). Overuse of fungicides in vineyards came at a cost. Several were shown to be phytotoxic to grapevines (Dias, 2012; Juang et al., 2012) and toxic to humans and the environment (Komárek et al., 2010), and some affected non-target organisms (La Torre et al., 2018), while others hampered beneficial insects (Thomson et al., 2000). In addition, the development of pathogen resistance pushed the search of ever more effective chemicals capable of dealing with increasingly resilient species (Verweij et al., 2009).

It was only in recent years, with the advent of molecular ecology and its tools, such as metagenomics and metabarcoding, that scientists have begun to understand how fungicides, along with herbicides and insecticides, impact the microbial ecology of environments with unpredictable medium- and long-term consequences (Komárek et al., 2010; Setati et al., 2012; Karlsson et al., 2014; Morrison-Whittle et al., 2017). The vineyard is an extremely diverse environment, characterized by complex interactions among plants, soil microbes, endophytes, and epiphytes, which are associated not only with grapevine health (Zarraonaindia and Gilbert, 2015; Jayawardena et al., 2018) but also with the concept of *terroir*, as it is a source of yeasts and bacteria especially important in winemaking (Pretorius et al., 1999; Setati et al., 2012).

Concerning plant health, grapevine trunk diseases (GTDs) are currently a major issue in viticulture, considered by some as “the new Phylloxera” due to their potential destructive power (Bruez et al., 2013). Early scientific reports on GTDs are over a century old (Surico, 2009), and the reasons underlying their recent worldwide outbreak have not yet been fully addressed. Unlike powdery and downy mildews agents (*Erysiphe necator* and *Plasmopara viticola*) and Phylloxera (*Daktulosphaira vitifoliae*), which were introduced from the Americas and found a susceptible host in *V. vinifera* (Jackson, 2014), GTD pathogens have presumably been co-evolving with grapevines for centuries, if not millennia (Mugnai et al., 1999).

Copper and sulfur fungicides were introduced approximately two decades before the first scientific reports on GTDs and the recent outbreak began some 30 years ago (Surico et al., 2004; Fontaine et al., 2016), approximately two decades after the massive introduction of synthetic broad-spectrum systemic fungicides in vineyards. To date, no study has investigated the effect of these fungicides, and that of other natural active ingredients, on the wood mycobiome and its possible implications in GTDs. To address this issue, we used metabarcoding (Illumina^®^ next-generation sequencing, NGS) and tested four hypotheses. (1) Are fungicides commonly sprayed in vineyards, to fight powdery and downy mildews, capable of affecting the resident wood mycobiome and/or (2) an inoculated consortium of fungal wood endophytes? (3) Is *Phaeomoniella chlamydospora*, a wood pathogen associated with several syndromes in grapevine (Surico 2009), affected by such fungicides, when artificially inoculated in wood? (4) Is the fungal consortium inoculation potentially exploitable in the control of early infections by *P. chlamydospora*, independently of fungicide treatments?

## Materials and Methods

### Experimental Setup

#### Plant and Fungal Material

The grapevines used in this assay were 1-year-old canes of cv. Cabernet Sauvignon, provided by the Viveiros VitiOeste nursery (Pó, Portugal), that were cut at three-bud-long size. The phytosanitary status of the propagation material was assessed by ensuring the absence of leaf symptoms and any visible wood symptomatology. The cuttings were rooted, potted in a mixture of peat and sand (1:1 v/v), and maintained under greenhouse conditions, at the temperature of 24 ± 5°C day/18 ± 5°C night.

The pathogenic ascomycete tested in this study was *P. chlamydospora* (CBS 161.90), from the CBS culture collection (Westerdijk Fungal Biodiversity Institute, Netherlands). Four grapevine endophytes, isolated from asymptomatic grapevine wood (cv. Cabernet Sauvignon), were used to produce the fungal consortium named “ACEA1,” namely, *Alternaria* (*Al.*) *alternata* A101, *Epicoccum nigrum* E279, *Cladosporium* sp. C22, *Aureobasidium* (*Au.*) *pullulans* AU86. All fungi were maintained in Petri dishes with vents, on potato dextrose agar medium (Difco^™^), at 25°C, in the dark.

#### Inoculum Preparation and Delivery

Four different inocula were prepared for delivery into the plant wood, three of them consisting of a suspension of conidia and/or fungal cells and the fourth of a control. Inoculum (i) consisted of a conidia suspension of *P. chlamydospora*, which was prepared by flooding a 2-week-old colony. The conidia were dislodged from the mycelium with a sterile glass rod, and the suspension was filtered through a double layer of cheesecloth. The conidial concentration was determined using a hemocytometer and adjusted to 1 × 10^5^ conidia per milliliter with sterile distilled water (SDW). Inoculum (ii) consisted of ACEA1, which was prepared by joining conidia suspensions of ascomycetes *Al. alternata*, *E. nigrum*, *Cladosporium* sp., and cells of yeast *Au. pullulans*. Cultures growth conditions and conidia/cell suspension preparation occurred similarly to inoculum (i), where the final concentration of each one of the four fungi was 1 × 10^5^ (conidia/cells) per milliliter. Inoculum (iii) was prepared by joining conidia suspensions of *P. chlamydospora* and ACEA1, in order to produce a solution with a final concentration of 1 × 10^5^ (conidia/cells) per milliliter of each one of the five fungi. Inoculum (iv) consisted of SDW.

**Figure 1:**
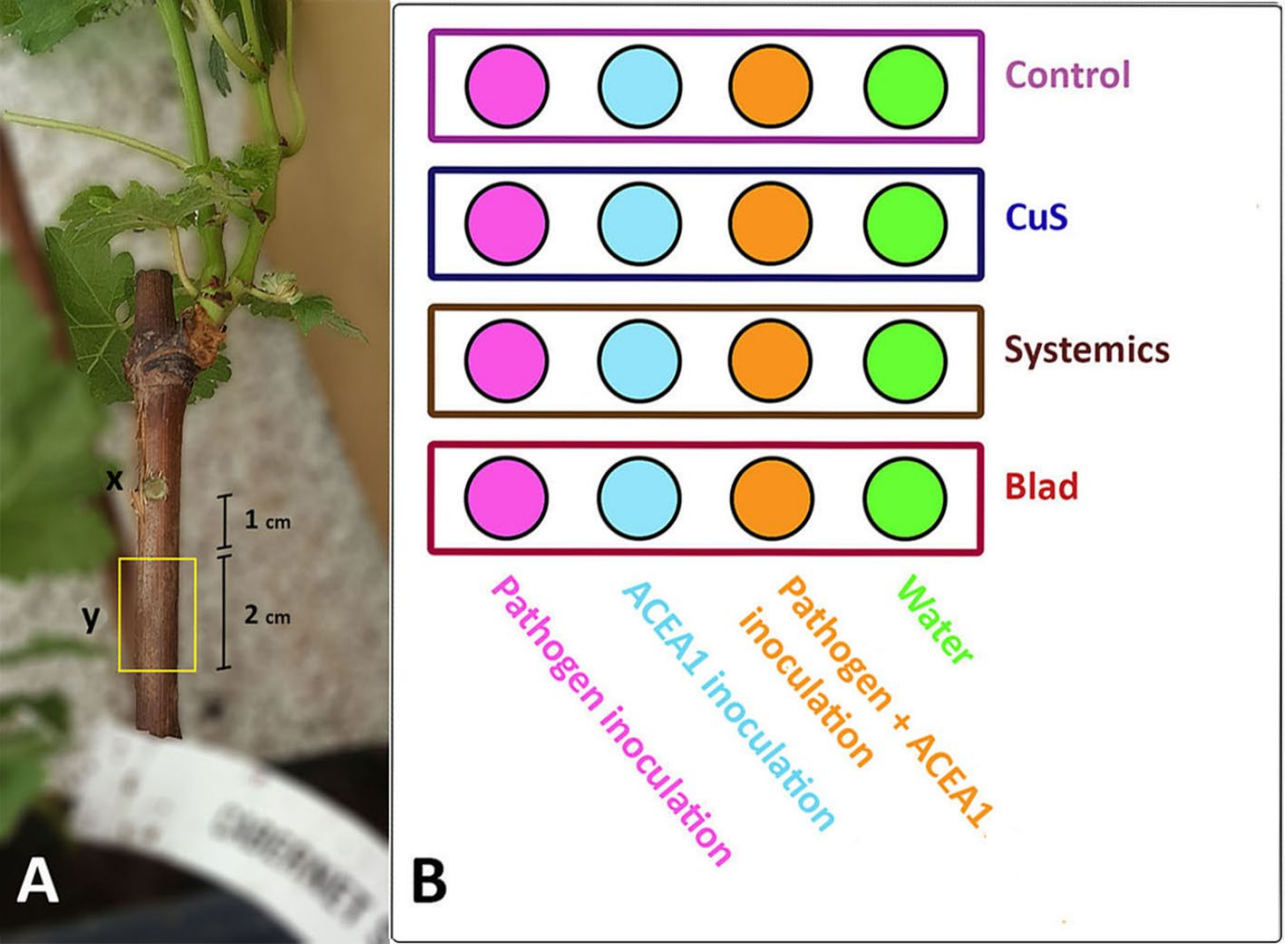
**(A)** Grapevine rooted cutting of cv. Cabernet Sauvignon. (*x*) Inoculation point of a conidia suspension of *Phaeomoniella chlamydospora*, ACEA1, *P. chlamydospora* and ACEA1, or sterile distilled water. (*y*) Two centimeters of wood, located 1 cm below the inoculation point, was sampled 3 months post-inoculation, and the wood’s mycobiome was examined through metabarcoding (*n* = 5 for each combination inoculum/treatment). **(B)** Experimental setup. Each circle represents a set of eight plants that underwent the same treatment. Same colors in the circles represent the same inoculum type, while rectangles represent different treatments. All possible combinations of inoculum and fungicide application by foliar spray are shown.

The delivery of the inocula into the rooted plants was performed as follows. Grapevine cuttings were surface disinfected with 70% (v/v) ethanol, and a wound in the wood was produced with the aid of a cork borer (4 mm diameter, 4 mm deep), approximately 1 cm below the green shoot (**Figure 1**). The four inocula were delivered with a micropipette by depositing 50 µl of solution in freshly made wounds, which were immediately sealed with Parafilm^®^. Each inoculum was delivered in 32 plants (**Figure 1**).

#### Treatments *via* Foliar Spray

Four treatments were tested for each inoculum type. The chemicals used and their application rate are reported in **Table 1**. The spray occurred every fortnight, starting from the seventh day post-inoculation and consisting in a total of six applications per treatment. Plants were sprayed on leaves and stem, with the exception of the control, which was sprayed exclusively on leaves. The chemicals were prevented from dripping onto the soil. All possible combinations of fungal inoculum and foliar treatment were tested, for a total of 16 combinations, each one consisting of eight biological replicates (**Figure 1**). The assay ended 1 week after the last spray (99 days post-inoculation).

**Table 1:** Chemical treatments sprayed fortnightly on grapevine rooted cuttings. Six applications were performed during 3 months. Each active ingredient was sprayed separately.

Treatment	Active ingredient	Trade name	Manufacturer	Formulation	Tested concentration
Control	Potassium permanganate^a^	Permanganato de potássio basi	Laboratórios Basi	Capsules 500 mg	1.0 g/L
Copper–sulfur	Copper oxychloride^b^	Cuprocol^®^	Syngenta	Copper oxychloride 36.5%	2.5 ml/L
	Sulfur^c^	Microthiol Special Disperss	Epagro	Sulfur wettable powder 80%	6.0 g/L
Systemic fungicides	Fosetyl-aluminum^b^	Aliette Flash^®^	Bayer	Fosetyl-aluminum 80%	2.5 g/L
	Penconazole^c^	Topaze^®^	Syngenta	Penconazole 10.5%	35 ml/100 L
Blad	Blad-containing oligomer^a^	Fracture^®†^	CEV/CONVERDE	Blad 20%	2.0 ml/L
				Other ingredients 80%	

^a^Treatments that target simultaneously powdery and downy mildews pathogens. ^b^Treatments that target downy mildew. ^c^Treatments that target powdery mildew.

^†^Supplier: FMC Corporation, Agricultural Products Group, 175 Market Street, Philadelphia, PA 19103, USA. EPA Reg. No. 84876-1-279.

Potassium permanganate (KMnO_4_) was chosen as control treatment due to the need of dealing with natural infections of powdery and downy mildews, which normally occur under greenhouse conditions. This chemical is effective against these pathogens due to its strong oxidative properties (La Torre et al., 2004), and we used the assumption that, when sprayed exclusively on leaves, it would not interact in a significant way with the wood mycobiome. Concerning treatments “Copper-Sulfur” and “Systemic fungicides,” the two active ingredients of each treatment were sprayed approximately 3 h apart from one another.

### Assessment of the Plant Health Status

**Figure 2:**
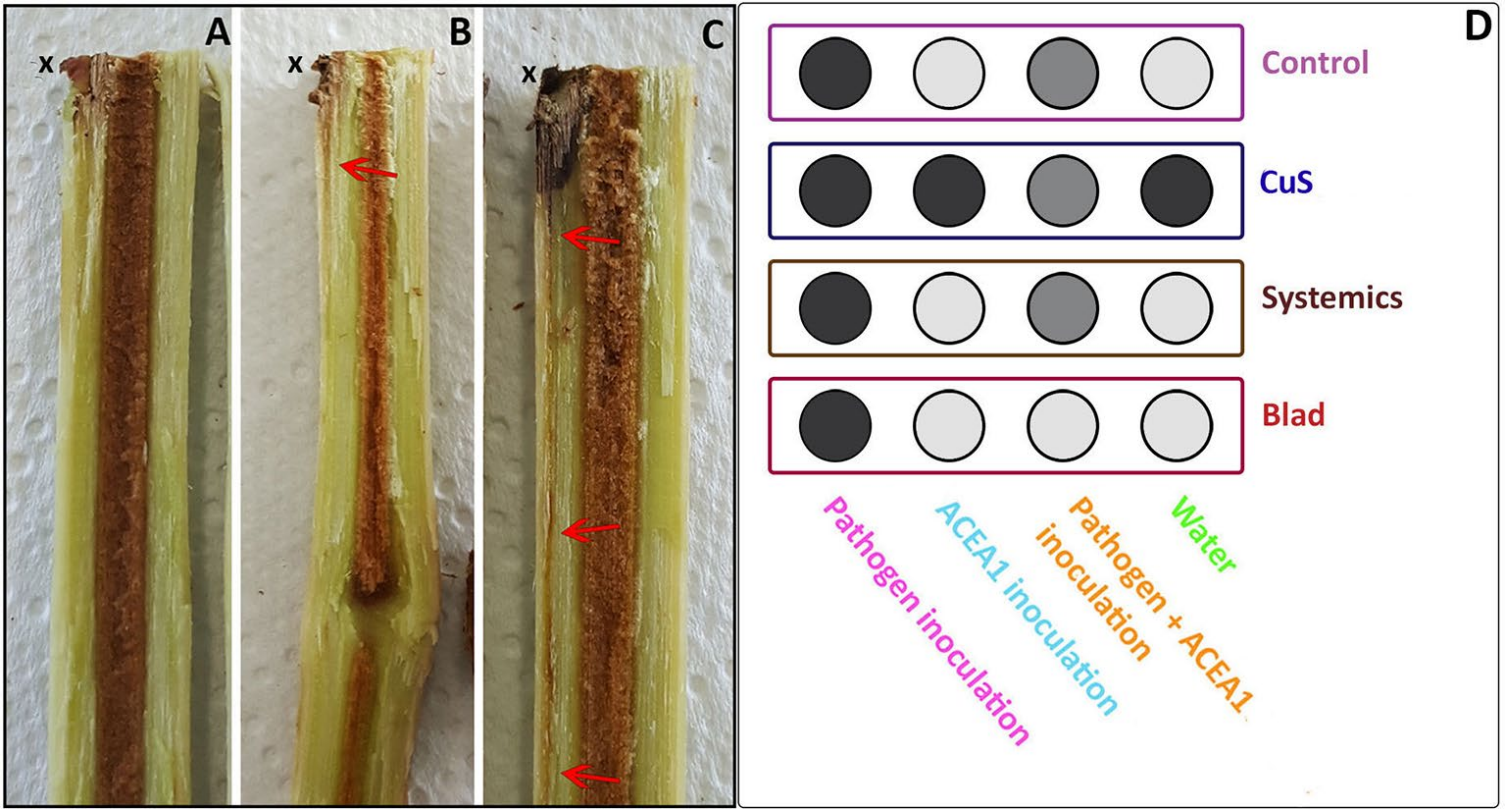
Grapevine rooted cuttings were examined, revealing different degrees of brown wood streaking symptomatology. Category 1, absence of symptoms **(A)** departing from the inoculation point (x) downwards; Category 2, presence of mild symptomatology **(B)**; Category 3, extensive symptomatology **(C)**. **(D)** Each category is presented in a different scale of gray, as an average of the observations for each treatment/inoculum (*n* = 3), where Category 1 is shown in light gray, Category 2 in gray, and Category 3 in dark gray.

Three parameters were evaluated to assess the health status of the plants at the moment of sampling. First, a visual inspection of the leaves and green shoots to establish whether any symptomatology related to the pathogen or ACEA1 inoculation manifested (*n* = 8 for each combination inoculum/treatment). Second, the shoot length was recorded, to understand if inoculated fungi and/or treatments had an effect on the growth of the plants (*n* = 8 for each combination inoculum/treatment). Lastly, for each combination of inoculum/treatment, three vines were subject to a destructive inspection to assess the presence and degree of internal wood symptomatology (e.g., brown streaking) visible in the stem of plants and starting from the inoculation point. Three categories of symptomatology degree were established: Category 1, absence of wood symptoms; Category 2, presence of brown streaking in proximity of the inoculation point; and Category 3, presence of brown streaking that extended several centimeters from the inoculation point (**Figure 2**).

### Wood Sampling, DNA Extraction, and Amplification

Grapevines were uprooted and the stem processed under laboratory conditions (*n* = 5 for each combination inoculum/treatment). The bark was sterilized by dipping the stem in 70% (v/v) ethanol followed by flame sterilization and then removed with the aid of a sterile scalpel. Exactly 2 cm of wood was sampled, 1 cm below the inoculation point (**Figure 1**). Samples were frozen, freeze-dried, and stored at −80°C.

Wood samples were ground to dust using sterile mortars and pestles, aided by liquid nitrogen. An aliquot of ground wood (0.25 ± 0.01 g) of each sample was added to DNA extraction columns (FastDNA^™^ SPIN Kit for Soil, MP Biomediacals^®^ LLC), and total DNA was extracted as described by the kit manufacturer. Three negative controls of the DNA extraction (no template) were included in this step and underwent the DNA extraction procedure.

The amplicon chosen for the metabarcoding analysis targets the internal transcribed spacer ITS2 region, and the primer set selected was ITS86F and ITS4R (Op De Beeck et al., 2014). For building libraries, we used a double-step PCR approach as reported by Feld et al. (2016). The full primer sequences including Illumina overhangs are ITS86F (5′-TCGTCGGCAGCGTCAGATGTGTATAAGAGACAG-GTGAATCATCGAATCTTTGAA-3′) and ITS4R (5′-GTCTCGTGGGCTCGGAGATGTGTATAAGAGACAG-TCCTCCGCTTATTGATATGC-3′).

Every first PCR contained 12.5 µl of Supreme NZYTaq II 2x Green Master Mix^™^ (NZYtech^™^), 0.5 µl of forward and reverse primers from a 10 µM stock, 1.5 µl of sterile water, and 5 µl of template. Each reaction was pre-incubated at 95°C for 2 min, followed by 40 cycles of 95°C for 15 s, 55°C for 15 s, and 72°C for 40 s; a further extension was performed at 72°C for 10 min. Negative controls of the PCRs were included alongside the samples. No-template DNA extracted samples also underwent the library preparation procedure.

A second PCR step for barcoding, MagBio bead fragment purification, and Qubit quantification were performed as reported in Gobbi et al. (2018). Final pooling was performed at 10 ng per sample. DNA sequencing was performed using an in-house Illumina MiSeq instrument and 2 × 250 paired-end reads with V2 Chemistry.

### Bioinformatics

After sequencing, demultiplex was performed using an Illumina MiSeq platform, and the raw data were analyzed using QIIME 2 v. 2018.2 (Caporaso et al., 2010), using the same pipeline described in Gobbi et al. (2018); denoised reads were quality-trimmed by 15 bp on the left to remove adapters and primers by using the quality filter plugin implemented in QIIME 2 (Bokulich et al., 2013). Filtered reads were then analyzed using DADA2 with the amplicon sequence variant (ASV) methods (Callahan et al., 2017). To minimize barcoding noise, operational taxonomic unit (OTU) counts below 25 reads were filtered out across all samples. Taxonomic assignments were performed at 99% identity using QIIME feature-classifier classify-sklearn with a naïve Bayes classifier trained with UNITE (Nilsson et al., 2013) v. 7.2 for ITS. After taxonomy assignment, the dominant features assigned to high taxonomical ranks such as order, class, or family were further investigated using BLAST to refine the analyses (Bokulich et al., 2018). The raw data of this study are available in the European Nucleotide Archive (ENA accession number PRJEB32853).

### Data Analysis

Shoot length data were subjected to analysis of variance (two-way ANOVA) to evaluate the effects of factors “fungicide treatment” and “inoculum type.” Significant means were compared using the Tukey *post hoc* test at a 5% significance level (GraphPad Prism 7.05).

The frequency table and its taxonomy table were combined, converted to biom format in QIIME (Caporaso et al., 2010), then merged with a table of metadata into an S4 object, and analyzed in R (v. 3.4.3) using the following packages: phyloseq, v. 1.22.3 (McMurdie and Holmes, 2013); vegan, v. 2.5.2 (Oksanen et al., 2007); DeSeq2 v. 1.22.1 (Love et al., 2014); ggplot2, v. 3.0.0 (Wickham, 2016); metacoder, v. 0.2.1.9005 (Foster et al., 2017); adespatial, v. 0.1.1 (Dray et al., 2018); data.table, v. 1.10.4.3 (Dowle and Srinivasan, 2017). R code is publicly available at github.com/Marieag/EMG.

The alpha diversity was measured using the Shannon diversity index and Pielou’s evenness and tested with one-way ANOVA with Tukey’s honestly significant difference (HSD) *post hoc* to determine differences among treatments and inoculum types.

We analyzed the beta dispersion to measure between-sample variances in abundance, computing average distances of the individual samples. The resulting ordination was plotted using non-metric multidimensional scaling (NMDS) combined with a Jaccard index matrix. To assess overall inter-group variance, we performed a PERMANOVA, using a Jaccard distance matrix with 999 permutations. The *post hoc* test for the PERMANOVA (performed on the ASV counts) was done using the adonis function from the vegan package in R.

To investigate any systematic changes between treatments, we calculated the differential abundance of any taxon with a relative abundance (RA) greater than 0.1% using DESeq2. Twenty-four pairwise analyses were run for every combination of fungicide treatment per inoculum type; log2 fold changes were calculated for each taxon resolved to species or genus level and tested for significance using a Wald test on the negative binomial distribution of the pairwise treatments. The *P*-value threshold was set to 0.05, and log fold change threshold to 0.1. In order to illustrate the results and to factor in the RA of unresolved species, we created differential heat trees using MetacodeR. A Wilcoxon rank sum test was applied to test differences between the same species in different treatments, and the resulting *P*-values were corrected for multiple comparisons using FDR, as implemented in MetacodeR. *P*-value threshold was set to 0.05.

## Results

### Evaluation of Plant Health Parameters

Visual inspection of grapevine cuttings, shoots length measurements, and wood examination occurred on the day of sampling. The visual inspection revealed no foliar symptomatology attributable to the wood pathogen *P. chlamydospora* or to the inoculated fungal consortium ACEA1.

**Figure 3:**
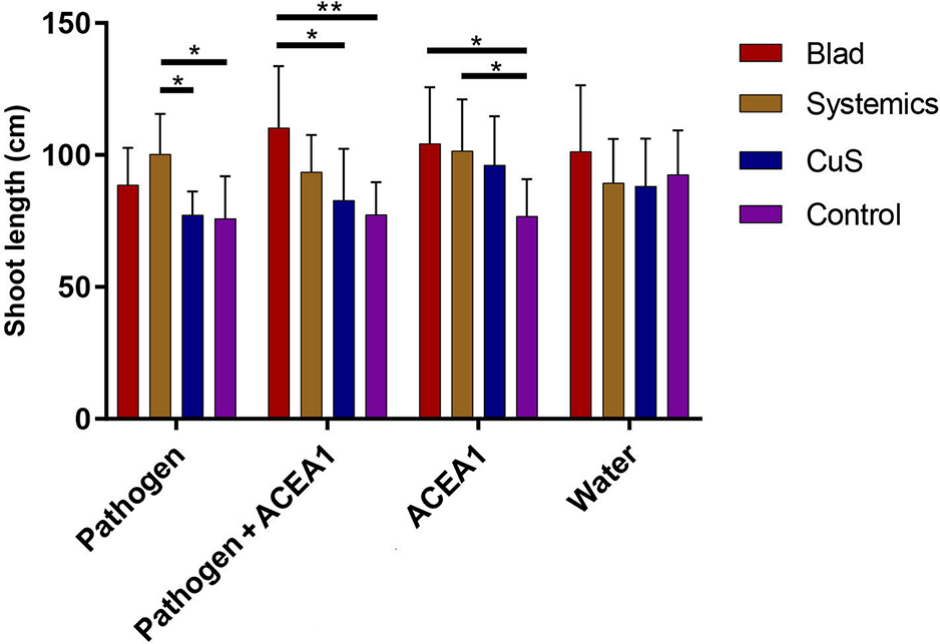
Barplot showing the measurements of grapevine shoot length. Error bars represent the standard deviation. Grapevines were inoculated with water, *Phaeomoniella chlamydospora* (Pathogen), a consortium of wood endophytes (ACEA1), and a combination of both (Pathogen + ACEA1). Grapevines were also treated with a foliar spray of potassium permanganate (Control), copper oxychloride and sulfur (CuS), and fosetyl-Al and penconazole (Systemics), Blad. *n* = 8 for each combination inoculum/treatment. Statistical differences, according to Tukey’s *post hoc* test, are shown by asterisk, where *P 0.05 and **P 0.01.

The statistical analysis of the green shoot length measurements, performed as a two-way ANOVA, revealed no significant differences for the factor “inoculum type” (*P* = 0.190), meaning that shoot growth was not influenced by inoculation with the pathogen, ACEA1, or the combination of both. However, a positive effect was present when comparing the factor “fungicide treatment” (*P* < 0.01), revealing that Blad and/or systemic fungicides increased the shoot growth of inoculated plants (pathogen, ACEA1, and pathogen + ACEA1), when compared with one or both the two other treatments (**Figure 3**). No significant differences were detected from the interaction between the two factors under examination (*P* = 0.136).

The examination of the wood revealed different degrees of symptomatology, ranging from absence of symptoms to extensive brown streaking departing from the inoculation point, as shown in **Figure 2**. Plants inoculated with *P. chlamydospora* presented extensive wood discoloration (Category 3; **Figures 2C, D**), under all spray treatments, while mild to absent symptoms, such as those shown in **Figures 2A, B** were recorded when *P. chlamydospora* was inoculated along with ACEA1. Plants inoculated with only water or ACEA1 did not show brown wood streaking symptoms, with the exception of the copper–sulfur treatment, suggesting that either copper oxychloride or sulfur or the combination of both may contribute to this plant response, independently from the inoculation of the pathogen.

### Sequencing Dataset Description

The dataset containing the NGS data produced and analyzed in this study accounts for 78 samples. These samples are represented by 6,454,075 high-quality reads distributed in 506 unique features. The average number of reads per sample is 82,744, which allowed an adequate sequencing depth to unravel the complexity of the grapevine cuttings wood mycobiome (alpha-rarefaction curves obtained with Shannon and observed OTUs are shown in **Figure S1**, results reported in **Table S2**). The raw ASV table (read counts) is available in **Table S3**.

Using BLAST to refine the taxonomic classification of the taxa composing ACEA1, taxon “Pleosporales incertae sedis” was assigned to *E. nigrum* in 98.12% of the reads. *Alternaria* sp. was retrieved in 16 sequence variants, with the dominant feature corresponding to 97.88% of the total. After blasting this dominant sequence, the assignment was refined to *Al. alternata*, with 100% identity. The genus *Cladosporium* sp. appears 12 times in our database for a total of 61,288 high-quality reads. Blasting the four dominant features, which cover 94.7% of the total amount of reads, they were all assigned to *Cladosporium* sp.

### A Qualitative Overview of the Wood Mycobiome

The richness of fungal taxa, identified examining all grapevine cuttings (*n* = 78), amounts to 66 species or genera of both ascomycetes and basidiomycetes. Among these 66 taxa, 30 of them are represented in an RA greater than 0.1%, while the remaining 36 taxa are considered rare taxa (RA < 0.1%; **Tables 2** and **S1**). Among the 30 most abundant taxa, several genera of ascomycetes are known to be involved in GTDs (e.g., *Neofusicoccum*, *Diaporthe*, *Cadophora*, and *Fusarium*), while no known pathogenic basidiomycete was detected (**Table 2**). The most frequent (non-inoculated) taxa are *Debaryomyces* sp., *Cryptococcus* sp., *Malassezia restricta*, *Malassezia globosa*, *Diaporthe* sp., *Acremonium alternatum*, *Candida sake* and *Candida friedrichii*. Sixteen out of the 30 most abundant taxa (**Table 2**) were detected in less than 10% of the total number of vines, and among these 16, eight of them were present only in a single rooted cutting. This observation highlights the high variability often encountered when comparing individual plants.

**Table 2:** Taxonomic classification of the most abundant taxa identified to genus or species level in the wood of grapevine rooted cuttings.

Phylum	Family	Species
Ascomycetes	-not available-	*Circinotrichum maculiforme* ^†^
	*Botryosphaeriaceae*	*Neofusicoccum* sp.*^†^
	*Cucurbitariaceae*	*Pyrenochaeta* sp.^†^
	*Davidiellaceae*	*Cladosporium* sp.
		*Cladosporium sphaerospermum*
	*Debaryomycetaceae*	*Meyerozyma guilliermondii* ^†^
	*Diaporthaceae*	*Diaporthe* sp.*
	*Didymellaceae*	Pleosporales incertae sedis (*Epicoccum nigrum*)
	*Dothioraceae*	*Aureobasidium pullulans*
	*Helotiales*	*Cadophora* sp.*^†^
	*Herpotrichiellaceae*	*Phaeococcomyces nigricans* ^†^
		*Phaeomoniella chlamydospora* *
	*Hypocreales*	*Acremonium alternatum*
		Ilyonectria destructans*^†^
	*Nectriaceae*	*Fusarium* sp.*^†^
	*Pleosporaceae*	*Alternaria* sp. (*Alternaria alternata*)
	*Saccharomycetaceae*	*Debaryomyces* sp.
		*Candida friedrichii*
		*Candida parapsilosis* ^†^
		*Candida sake*
		*Candida tropicalis* ^†^
Basidiomycetes	*Cystofilobasidiaceae*	*Cystofilobasidium capitatum* ^†^
	*Filobasidiaceae*	*Naganishia* sp.^†^
	*Malasseziaceae*	*Malassezia* sp.^†^
		*Malassezia restricta*
		*Malassezia globosa*
		*Malassezia sympodialis* ^†^
	*Peniophoraceae*	*Peniophora* sp.^†^
	*Sporidiobolaceae*	*Sporidiobolus* sp.^†^
	*Tremellaceae*	*Cryptococcus* sp.

The relative abundance of the listed taxa was equal to or greater than 0.1% of the total dataset. The taxonomic classification of species between parentheses was attributed using BLAST.

*Taxa associated with grapevine trunk diseases.

^†^Taxa detected in less than 10% (n = 8 or less) of the total number of vines (n = 78).

The metagenomic analysis detected *P. chlamydospora* in 28% of non-inoculated plants (RA > 0.1%), while the presence of wood endophytes that composed ACEA1, in non-inoculated grapevines, was revealed to be very low. *Alternaria* sp. and *Cladosporium* sp. were present in 8% of the plants, while *E. nigrum* (Pleosporales incertae sedis) and *Au. pullulans* in 3% of them (RA > 0.1%).

### Alpha Diversity

**Figure 4:**
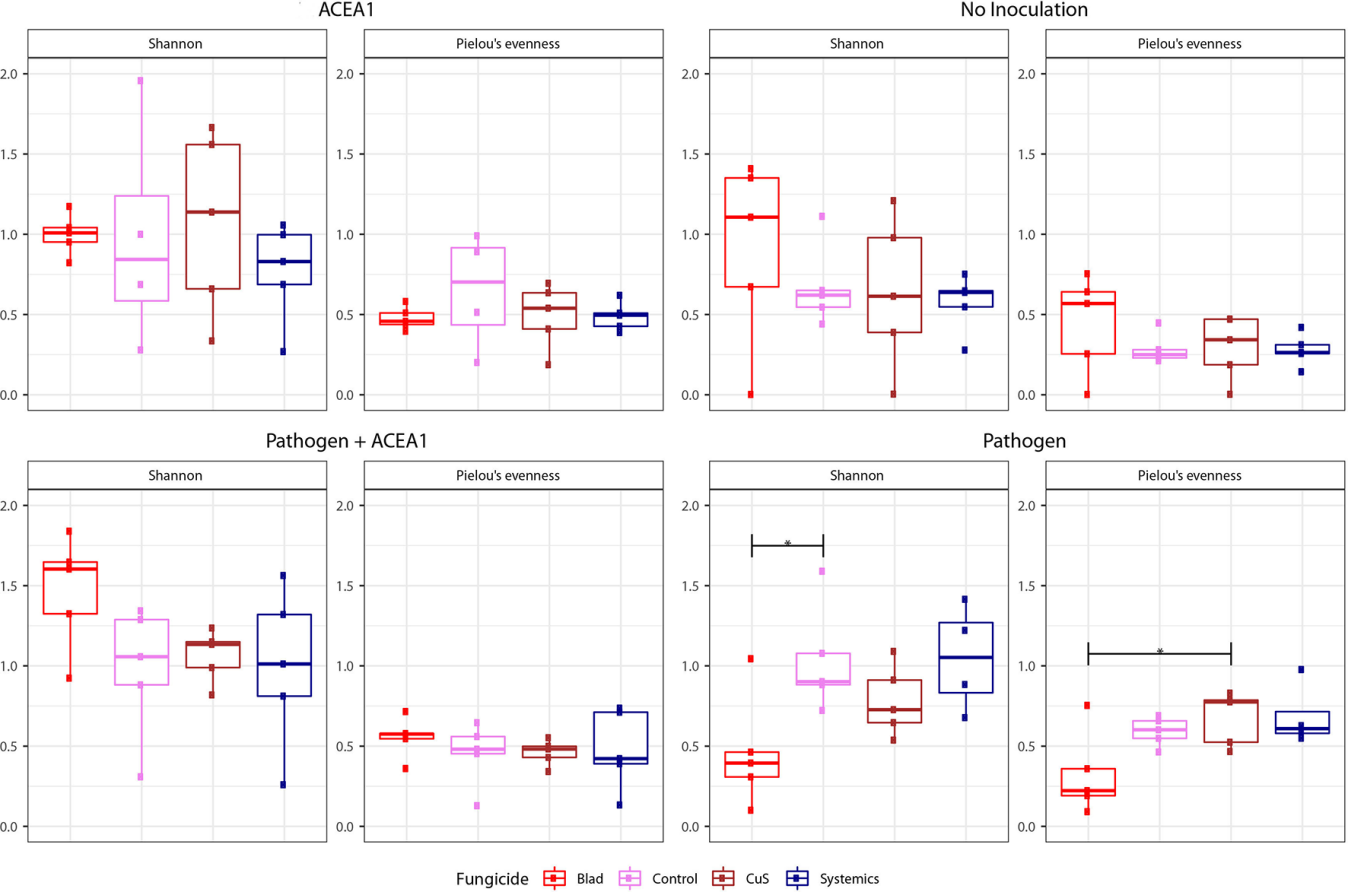
Box plots of diversity indexes (Shannon, Pielou’s evenness) of the fungal community present in grapevine cuttings added with water (No inoculation) or inoculated with *Phaeomoniella chlamydospora* (Pathogen), a consortium of fungal wood endophytes (ACEA1), or both *P. chlamydospora* and ACEA1. Inoculated plants were sprayed with either Blad or potassium permanganate (Control) or copper oxychloride and sulfur (CuS) or penconazole and fosetyl-aluminum (Systemics). *n* = 5 for each combination inoculum/treatment. The black, horizontal brackets at the top of the figures denote statistical comparisons of the two treatments at each end of the bracket, calculated using one-way ANOVA with Tukey’s honestly significant difference (HSD) *post hoc*. Statistical differences are shown by asterisk, where *P 0.05.

The boxplots in **Figure 4** show the Shannon diversity and Pielou’s evenness in grapevines treated with different fungicides and inocula. The Shannon diversity and evenness of the resident mycobiome of non-inoculated plants did not vary in relation to the fungicide treatment, according to Tukey’s HSD (*P* > 0.05). On the other hand, the inoculation of *P. chlamydospora* comported changes in both diversity and evenness (*P* < 0.05) of the mycobiome, when vines were sprayed with Blad (**Figure 4**). This active ingredient induced a reduction in Shannon diversity when compared to control-treated plants, as well as a strong trend when comparing Blad-treated plants to those treated with systemic fungicides (*P* = 0.058). No diversity differences were significant among other treatments (*P* > 0.05). The evenness of the fungal communities significantly varied when comparing Blad-treated vines with copper–sulfur-treated vines (**Figure 4**), and a strong trend was detected when comparing the former treatment with systemic fungicide-treated vines (*P* = 0.051). The application of the different fungicides did not affect the Shannon diversity and evenness of vines inoculated with either ACEA1 alone or ACEA1 and the pathogen (*P* > 0.05).

### Beta Dispersion

**Figure 5:**
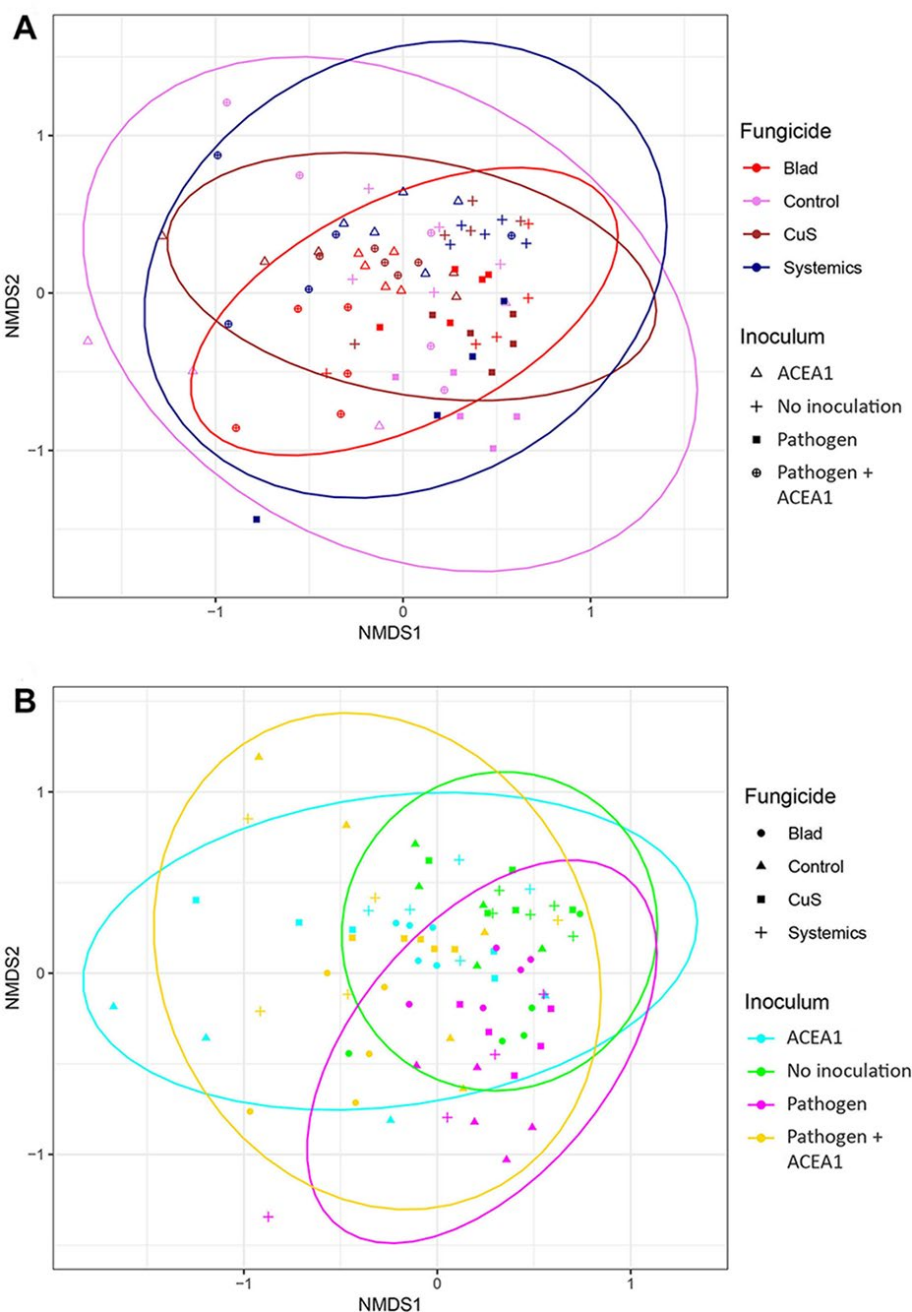
Non-metric multidimensional scaling (NMDS) plots based on Jaccard’s index. Fungal communities present in grapevine cuttings added with water (No inoculation), *Phaeomoniella chlamydospora* (Pathogen), a consortium of fungal wood endophytes (ACEA1), or both *P. chlamydospora* and ACEA1. Inoculated plants were sprayed with either Blad or potassium permanganate (Control) or copper oxychloride and sulfur (CuS) or penconazole and fosetyl-aluminum (Systemics). *n* = 5 for each combination inoculum/treatment. Ellipses illustrate the multivariate normal distribution of samples within the same fungicide **(A)** or inoculum **(B)** group.

The Jaccard index, when visualized in an NMDS plot, shows a considerable overlap for different fungicide treatments and inoculum types (**Figure 5**). The PERMANOVA indicates a significant difference between groups (*P* = 0.001), but looking at the ordination, the difference seems to lie in the clustering of the observations, rather than any distinct difference in sample composition. For example, the fungal communities in Blad-treated plants cluster more tightly than the control-treated ones, and a similar trend applies to non-inoculated vines when compared with those inoculated with both pathogen and ACEA1.

### Effect of Inoculum Type and Fungicide Treatments on Taxa Abundance

**Figure 6:**
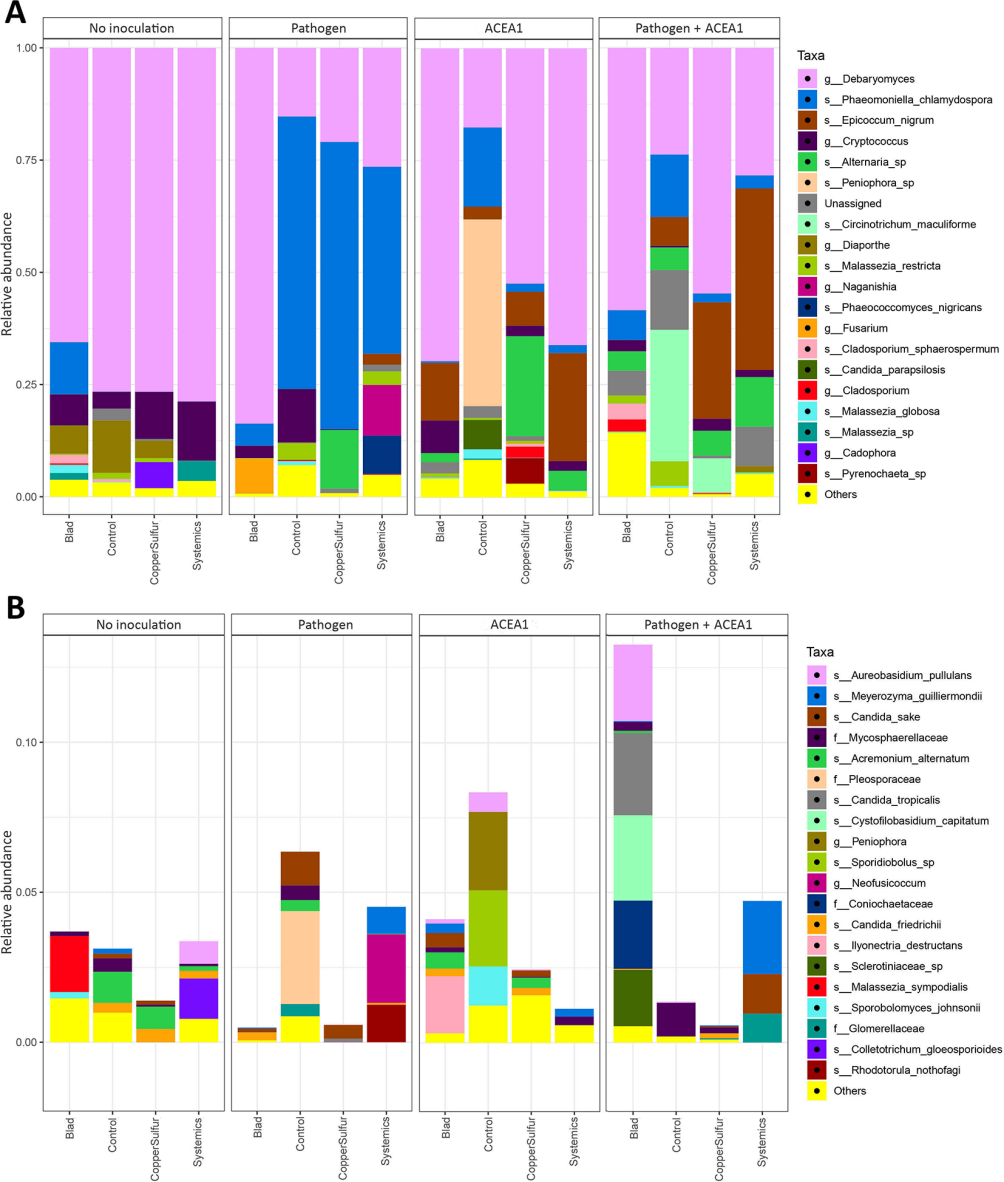
Barplots of the relative abundance of the 20 most abundant taxa **(A)** identified to species (s_), genus (g_), or family (f_) level, found in rooted grapevine cuttings non-inoculated (No inoculation) or inoculated with *Phaeomoniella chlamydospora* (Pathogen) or a consortium of fungal wood endophytes (ACEA1) or a combination of both (Pathogen + ACEA1). Grapevines were treated with either Blad or potassium permanganate (Control) or copper oxychloride and sulfur (CopperSulfur) or fosetyl-aluminum and penconazole (Systemics). *n* = 5 for each combination inoculum/treatment. “Unassigned” are taxa identified to a lower taxonomic level than family or non-identified, “Others” are taxa not included in the 20 most abundant. **(B)** The 20 most abundant taxa within the “Others” group of **(A)** are shown.

**Figure 7:**
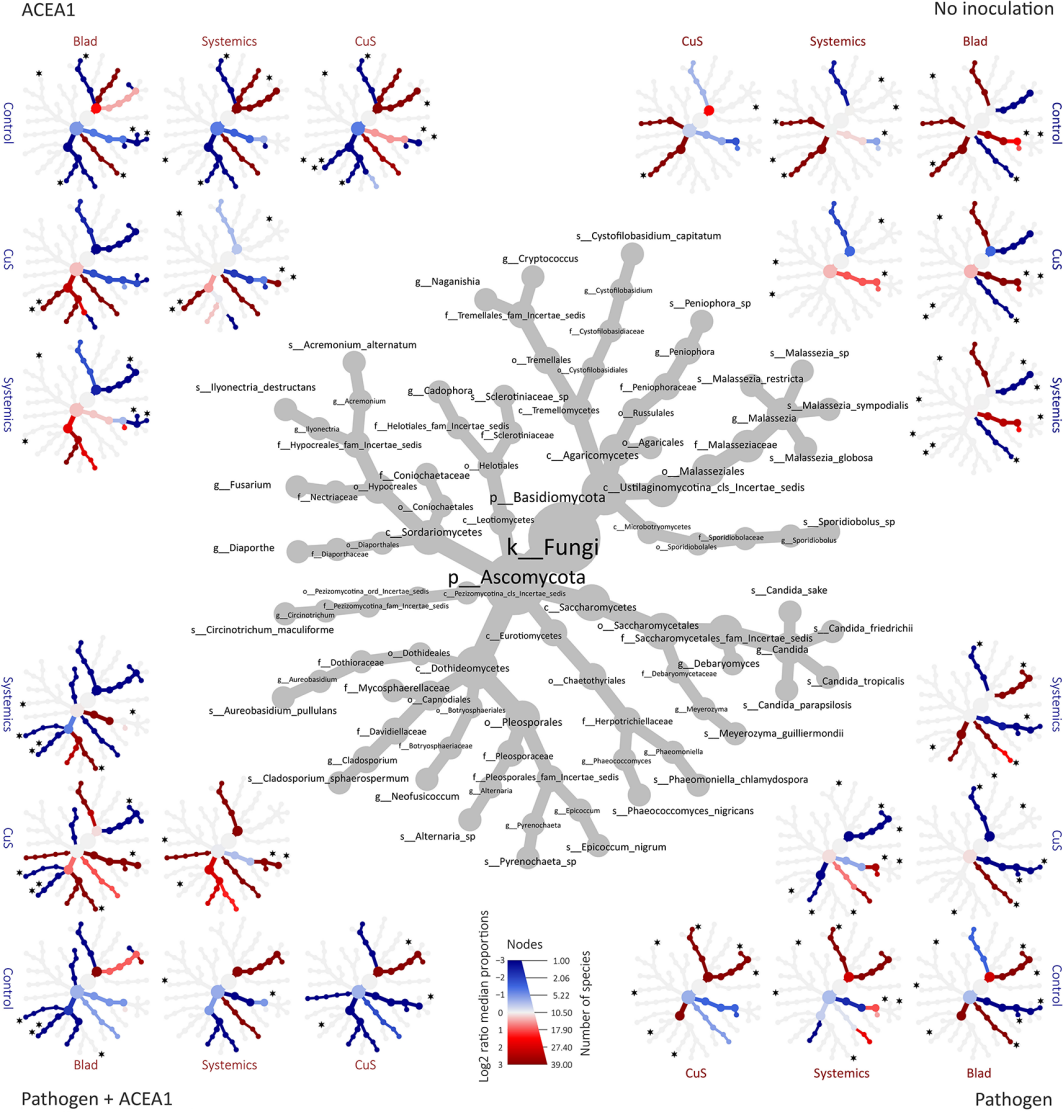
Differential heat tree matrixes depicting the change in taxon abundance between different fungicide treatments, represented in the dataset with a relative abundance (RA) > 0.1% (*n* = 5 for each combination inoculum/treatment). The size of the individual nodes in the gray cladogram depicts the number of taxa identified at that taxonomic level. The smaller cladograms show pairwise comparisons between each treatment, with the color illustrating the log2 fold change: a red node indicates a lower abundance of the taxon in the treatment group stated on the abscissa than in the treatment group stated on the ordinate. A blue node indicates the opposite. A black star next to a node represents the statistical differences according to DeSeq2 (*P* 0.05), for the most frequent taxa (**Table 2**).

When examining the effect of fungicide treatments on the fungal communities of both non-inoculated and inoculated plants, no differences are detected according to the Wilcoxon test (*P* > 0.05). However, several taxa were found to have significantly different abundances between treatments according to the Wald test on the pairwise treatment comparisons, and the combined MetacodeR and DESeq analyses revealed overrepresentation and underrepresentation of numerous taxa under different treatments (**Figures 6** and **7**). Among the taxa identified as differently abundant, we find some of the fungi present in less than 10% of the total number of plants (**Table 2**). This low frequency suggests that the detected differential abundance may not lie in the effect of the treatments, but instead in the uneven distribution (presence/absence) of these taxa in individual vines. For this reason, these taxa are not going to be further examined.

In non-inoculated vines, *Candida sake* and *C. friedrichii*, present in control-treated plants, are not detected in Blad-treated vines; similarly, *C. sake*, *Malassezia Restricta*, and *M. globosa* are present in control-treated plants but absent in systemic fungicide-treated vines; *Diaporthe* sp. was detected under all treatments with the exception of systemic fungicides. None of the most frequent taxa differs significantly when comparing control- with copper–sulfur-treated grapevines (**Figure 7**).

In inoculated vines, we assumed that a successful colonization only occurred when inoculated fungi were detected in at least 50% of the biological replicates (per treatment/inoculation combination). The inoculation with *P. chlamydospora*, whether alone or in combination with ACEA1, resulted in a successful wood colonization, 1 cm below the inoculation point, in 87% of the cases. In the pathogen-only inoculation, fungicide treatments affected the RA of *P. chlamydospora* (**Figure 7**, **Table 3**) and that of other taxa. The foliar spray with Blad significantly decreased the RA of this fungus when compared with the control and other treatments (*P* < 0.05).

**Table 3:** Relative abundance of inoculated fungi in plants that were subject to different fungicide treatments.

	No inoculation				Phatogen inoculation				ACEA1 inoculation				Phatogen + ACEA1 inoculation			
	C	CuS	Sys	Blad	C	CuS	Sys	Blad	C	CuS	Sys	Blad	C	CuS	Sys	Blad
***Phaeomoniella chlamydospora***	–	–	–	11.6*	60.7*	64.0*	33.3*	4.9*	17.6	1.8	1.8	0.4	13.9*	2.0*	2.9	6.6*
***Alternaria alternata*** (*Alternaria* sp.)	–	–	–	–	–	13.1	–	–	–	22.3*	4.3*	2.1*	5.0	5.5*	11.0*	4.3*
***Epicoccum nigrum*** (Pleosporales incertae sedis)	–	–	–	–	–	–	2.0	–	2.8*	7.6*	24.1*	12.9*	6.4	25.9*	40.4*	–
***Aureobasidium pullulans***	–	–	0.8	–	–	–	–	–	0.7	–	–	0.2	–	–	–	2.5*
***Cladosporium*** **sp.**	–	–	–	0.3	0.3	–	0.1	–	–	2.5	–	–	–	0.2	–	2.7*

Grapevine cuttings were non-inoculated (No inoculation), inoculated with P. chlamydospora (Pathogen inoculation), or inoculated with a consortium of fungal wood endophytes (ACEA1 inoculation) or a combination of both (Pathogen + ACEA1 inoculation). Grapevines were treated with a control (potassium permanganate; C) or copper oxychloride and sulfur (CuS) or fosetyl-aluminum and penconazole (Sys) or Blad. Different colors represent different intervals of relative abundance (≤1; 1–5; 5–10; 10–20; >20). n = 5 for each combination inoculum/treatment.

(*) 50% or more of the biological replicates were successfully colonized by the inoculated fungus

In grapevines inoculated with ACEA1 and sprayed with potassium permanganate (control), only *E. nigrum* could successfully colonize the wood examined. The abundance of this ascomycete increased when plants were treated with Blad or systemic fungicides (**Table 3**). *Al. alternata* was absent (RA > 0.1%) in control-treated plants, while it was detected, in different abundances, under all other treatments (**Table 3**). The wood colonization by *Au. pullulans* was poor to absent, and it was never detected in 50% or more of the inoculated plants (**Table 3**). *Cladosporium* sp. was present exclusively in copper–sulfur-treated plants, although not detected in 50% or more of the inoculated plants (**Table 3**).

The simultaneous inoculation of *P. chlamydospora* and ACEA1 resulted in a lower abundance of the pathogen, when compared with the *P. chlamydospora*-only inoculum (**Table 3**), for all treatments except Blad. *Al. alternata* was detected under all fungicide treatments, except for the control-treated vines. *E. nigrum* exclusively colonized the wood when vines were treated with systemic fungicides or copper–sulfur. The Blad treatment facilitated the wood colonization by *Au. pullulans* and *Cladosporium* sp., which were not detected under any of the other treatments (**Figure 7**).

## Discussion

Current knowledge on the effects of fungicides on the grapevine microbiome is scarce. Perazzolli et al. (2014) examined the effect of penconazole and a biological control agent on the grapevine phyllosphere, finding that bacterial and fungal communities are only minimally affected by both treatments. However, studies that investigated the effect of vineyard management systems (organic or integrated pest management) on endophyte communities, discovered significant differences, in both fungi (Pancher et al., 2012) and bacteria (Campisano et al., 2014). These results suggest that fungicide treatments may be one of the factors driving such microbiome changes. In this study, we investigated the changes that occur in the wood mycobiome in response to the application of fungicides, sprayed on leaves, in young plants. Due to the numerous factors that may influence our understating of this interaction, we decided to perform the experiment under semi-controlled conditions (i.e., greenhouse) and with no experimental repetitions. It follows a brief list of factors known to play a role in shaping the wood mycobiome of grapevines.

- The age of the plants—Young grapevines (and canes) are known to host a lower richness in fungal taxa, when compared to adult vines (Dissanayake et al., 2018; Del Frari et al., 2019a).
- The management of the vineyard—Fungal endophytic communities found in organic vineyards can significantly differ from those found in vineyards that use an integrated pest management strategy (Pancher et al., 2012).
- The geographical location—In the same study by Pancher et al. (2012), significant differences in the wood mycobiome were detected when examining different vineyard locations.
- Grapevine cultivar—Jayawardena et al. (2018) proposed that grapevine cultivar, along with geographical location, is a determinant factor in the wood mycobiome composition. This observation is also supported by the results of a previous study by Travadon et al. (2016).
- Seasonality—In a study by Bruez et al. (2014), grapevine wood sampled in different seasons harbored diverse community structures.
- The physiological status of grapevines may also influence their susceptibility to infection (e.g., biotic and abiotic stress; Gramaje et al., 2018).

Considering all these variables, it is reasonable to forecast that if any of the above factors changes, our understanding of the effect of fungicides on the wood mycobiome may vary. For this reason, the results obtained in this study should be considered as a case study, rather than a general understanding, and further investigation is required.

In order to assess the effect of fungicides on the early colonization success by wood endophytes, we selected four non-pathogenic fungi that are frequently found in grapevine wood and that are believed to hold potential in the biological control of wood pathogens (Pancher et al., 2012; Mondello et al., 2017; Jayawardena et al., 2018; Pinto et al., 2018). The choice of using a consortium instead of single fungus springs from the two following ideas:

- To examine the effect of fungicide treatments on the early wood colonization success—The inoculation of multiple endophytes allows us to screen a higher number of fungi simultaneously.
- To examine the effect of simultaneously inoculating multiple endophytes and *P. chlamydospora*—The antagonistic interactions among endophytes and pathogen become more complex when compared to the confrontation between a single antagonist and a single pathogen. We hypothesized that the competition for space and nutrients, as well as the chemical war that occurs in the wood (e.g., competition-induced fungal metabolites; Hardoim et al., 2015), would strongly reduce the likelihood of *P. chlamydospora* producing a successful infection.

### Visual Inspection, Shoot Length, and Brown Wood Streaking

The absence of foliar symptoms in the vines inoculated with ACEA1 confirms the current understanding that none of the selected organisms composing ACEA1, when present in the wood, are pathogenic in grapevines (Jayawardena et al., 2018). Concerning *P. chlamydospora*, Sparapano et al. (2001) observed that this fungus induced foliar symptom appearance, albeit only several months/years post-inoculation; therefore, the absence of foliar symptoms at the end of the experiment was expected.

The differences in length of the green shoot, only observed in response to fungicide treatments, suggest that both systemic fungicides and Blad positively affect the growth of vines (**Figure 3**); however, their mechanisms of action remain to be assessed.

Wood discoloration, in the form of brown wood streaking, is a result of the oxidation and polymerization of phenolic compounds, through the action of phenolases, a plant response to pathogen infections (Agrios, 2012) but also to other stresses such as mechanical injuries (Pouzoulet et al., 2013). (i) The absence of brown wood streaking in water- and ACEA1-inoculated vines and in plants treated with potassium permanganate, systemic fungicides, and Blad confirms that none of the components of ACEA1 are pathogenic. On the contrary, copper–sulfur-treated plants manifested extensive brown wood streaking. A recent report revealed that wood necrosis caused by grapevine wood pathogen *Neofusicoccum parvum* was promoted by the presence of high doses of copper in soil (Bruez et al., 2017). Copper is also associated with fungal pigmentation production (Griffith et al., 2007), a virulence factor (e.g., fungal melanins; Jacobson, 2000); laccase synthesis, which is involved in wood degradation (Tychanowicz et al., 2006); and induction of phytotoxic effects, with changes in plant morphology, biochemistry, and physiology (Bruez et al., 2017). Overall, these clues suggest that copper may play a relevant role in the development of brown wood streaking, by interacting with plant and endophytes. (ii) The extensive symptomatology recorded in plants inoculated with *P. chlamydospora*, under all fungicide treatments, indicates that the plant readily responded to infection, and it did so independently of the abundance of *P. chlamydospora* in the wood (e.g., Blad treatment; **Table 3**). Wood pathogens are known to induce the appearance of brown wood streaking, but they are not always found in symptomatic wood, especially the farther from the inoculation point (Mugnai et al., 1999). This suggests that, under some circumstances, the extent of wood streaking may not be correlated with the abundance of the pathogen. Moreover, *P. chlamydospor*a has been previously reported in wood free from brown streaking symptoms (Rumbos and Rumbou, 2001; Abreo et al., 2011; Hofstetter et al., 2012), and its ecology requires further investigation. (iii) Moderate to absent wood symptoms were observed when co-inoculating *P. chlamydospora* and ACEA1, suggesting that the interactions among fungi resulted in a weaker or absent plant response to the infection. In fact, the reduction in wood streaking extent, due to the presence of fungal antagonists, has been previously reported in grapevines (Mondello et al., 2017; Del Frari et al., 2019b). It is worth remembering that the observations related to the brown wood streaking symptomatology made in this work are preliminary, and further evidence is necessary to assess the relation among wood inhabiting fungi–fungicides–wood symptomatology.

### Effect of Fungicides on the Resident Mycobiome and on the Early Colonization by Endophytes

Previous studies revealed that fungicide application can affect the mycobiome of grapevines and other crops (Karlsson et al., 2014; Morrison-Whittle et al., 2017), although none of them investigated the fungal communities present in the wood. This study shows that some fungal taxa composing the resident wood mycobiome of grapevine cuttings cv. Cabernet Sauvignon are affected by fungicide application (**Figure 7**). This observation suggests that active ingredients interacted with fungi either directly (e.g., copper oxychloride is known to be absorbed in the xylem of grapevines, when sprayed on the bark of vines; Di Marco et al., 2011) or indirectly, for example, stimulating plant defenses (García et al., 2003). Further studies are necessary to confirm this preliminary understanding, keeping into account (i) the variability in taxa composition that may characterize individual plants and (ii) that the DNA of dead fungal cells, supposedly affected by fungicides, may persist in the wood, during short-term experiments, hiding the magnitude of the treatment effect. It is also necessary to investigate whether the changes observed in taxa abundance are temporary or long-lasting, therefore determining the overall resilience of the endophytic communities that compose the resident mycobiome.

Multitrophic interactions involving plants and fungal and bacterial endophytes are still poorly understood (Hardoim et al., 2015). Current literature shows that host and microbe genotypes, as well as abiotic factors, can influence successful colonization by endophytes (Hardoim et al., 2015). In this study, we show that also fungicide application can affect the early colonization by fungal endophytes, in particular those composing ACEA1 (**Table 3**). Therefore, fungicides seem to have the potential of shaping the wood mycobiome by favoring/inhibiting the early wood colonization of specific fungi. For this reason, we propose to keep in consideration fungicide treatments when describing endophytic communities in grapevines.

### Effect of Fungicides on the Early Colonization by *P. chlamydospora*

Numerous trials have been conducted, over the last 20 years, in order to find reliable means to control infections by wood pathogens, including *P. chlamydospora*. Chemical control, in the form of foliar spray or endotherapy, led primarily to negative or moderate results, while more promising ones have been achieved through pruning wound protection (Mondello et al., 2017). In this study, the copper–sulfur and systemic fungicide treatments did not affect the colonization success of the pathogen, within the timings of the experiment (**Figure 6**). A preliminary study in support of the neutral effect of copper oxychloride on the colonization success of *P. chlamydospora* was presented by Di Marco et al. (2011), although no literature is available on the influence of sulfur or other synthetic fungicides. Blad was the only active ingredient capable of significantly inhibiting the wood colonization success of *P. chlamydospora* (Del Frari et al., 2018; Monteiro et al., 2015). This is the first report of a foliar spray capable of reducing the wood colonization success of this pathogen.

### Use of ACEA1 in the Biological Control of *P. chlamydospora*

As postulated, the wood colonization success of *P. chlamydospora* was greatly reduced when co-inoculated with a consortium of fungal wood endophytes (ACEA1) that are considered potential antagonists (**Figure 6**). Some of the possible explanations are found in the interactions that occur during conidia germination and mycelial growth (e.g., release of metabolites) and by increasing the competition for space and nutrients (Hardoim et al., 2015). Fungi composing ACEA1 are frequently isolated from grapevine wood; however, their exact niche (e.g., xylem vessels, tracheids) remains to be assessed. A histological examination of the wood may unveil the exact interaction that takes place among the components of ACEA1 and *P. chlamydospora*. Only minor changes in the abundance of the pathogen are observed with different fungicide treatments, making ACEA1 a potential tool to be exploited in biological control. Interestingly, the Blad treatment facilitated the wood colonization by both *Cladosporium* sp. and *Au. pullulans*, while inhibiting that of *E. nigrum*, in the presence of *P. chlamydospora*, when compared with the results obtained from ACEA1-only inoculation (**Table 3**). This observation suggests the occurrence of complex interactions in which not only the fungicide but also the pathogen play a primary role (e.g., competition-induced metabolites), resulting in selective wood colonization. Further investigation is necessary to confirm these results also from a quantitative point of view. It is also important to understand if the effect observed by the addition of ACEA1 is long-lasting, and it may be successfully applied to plants with an established infection, as a curative strategy.

## Conclusion

This study shows that some common fungicides that have been applied in viticulture for decades (copper–sulfur and systemics) affect the resident mycobiome and/or the wood colonization success of some endophytes, while leaving nearly unaffected the wood pathogen *P. chlamydospora*. This has unpredictable consequences, in both the medium and long terms, on the wood mycobiome ecology and its influence on grapevine well-being. It is reasonable to wonder whether the wood mycobiome composition of grapevines before the fungicide era was considerably different from that of today. As the diversity of a biological system is positively correlated with its stability (McCann, 2000), a loss in endophytic species that antagonize GTD pathogens, such as *P. chlamydospora*, may be a possible explanation for the recent success of these pathogenic fungi. Another explanation may lie in the fungicide-driven increased likelihood of infection by some fungi, which creates an imbalance in the stability of the plant–mycobiome biological system.

This work addressed only four endophytes, although the wood mycobiome of adult plants is known to be potentially colonized by hundreds of species, making this just the beginning of the research in this sense. In conclusion, enhancing the complexity of the wood mycobiome, for example, using multiple endophytic antagonists, may considerably increase our chances to control GTD-associated pathogens. While research on endophytic fungal consortia advances, Blad seems a promising active ingredient to confront early infections by *P. chlamydospora*, in young grapevines. This active ingredient is non-toxic for the environment and humans, has growth-promoting properties, and strongly antagonizes the wood colonization success of *P. chlamydospora*, making it a promising tool in viticulture.

## Data Availability Statement

The raw data of this study are available in the European Nucleotide Archive (ENA accession number PRJEB32853).

## Author Contributions

GF conceived the study, performed greenhouse experiment, performed wet-lab work, interpreted and discussed the results, and wrote the manuscript. AG performed wet-lab work, performed bio-informatics analyses, wrote the manuscript. MA performed statistical analyses and data visualization. HO, LH, and RF supervised the study and reviewed the manuscript.

## Funding

This study was funded by the Horizon 2020 Programme of the European Commission within the Marie Sklodowska-Curie Innovative Training Network “MicroWine” (grant number 643063 to G. Del Frari, A. Gobbi, L. Hestbjerg Hansen and R. Boavida Ferreira) and by Portuguese national fund FCT Unit funding UID/AGR/04129/2019 (LEAF).

## Conflict of Interest

The authors declare that the research was conducted in the absence of any commercial or financial relationships that could be construed as a potential conflict of interest.

## Supplementary Material

Table S1 Fungal taxa identified to genus or species level in the wood of grapevine rooted cuttings, at relative abundances (RA) inferior to 0.1 % or 0.01% of the total.

Table S2 Rarefaction curves results based on richness, calculated on the number of amplicon sequence variants (ASVs), and on Shannon index.

Figure S1 Rarefaction curves of alpha-diversity indexes; each line correspond to one sample in a range from 0 to 18000 reads. Each box plot represent the distribution of the selected alpha diversity metric for each sample at each 2000 reads of sampling depth. The lower and upper whiskers of the box plot correspond to the 9th and 91st percentiles of the distribution, while the lower and upper extents of the box correspond to the 25th and 75th percentiles of the distribution (respectively). The horizontal bar through the middle of the box is the median of the distribution (i.e., the 50th percentile). Outlier points of these distributions are not shown. The lines connect all the medians from the different. A) Rarefaction curves based on richness, calculated on the number of amplicon sequence variants (ASVs). B) Rarefaction curves based on Shannon index.
