# Supplementary material for: Fungicides and the Grapevine Wood Mycobiome: A Case Study on Tracheomycotic Ascomycete Phaeomoniella chlamydospora Reveals Potential for Two Novel Control Strategies
Source: Front Plant Sci. 2019 Oct 31;10:1405. doi: 10.3389/fpls.2019.01405 (PMC6836639; doi:10.3389/fpls.2019.01405)
Supplement: Table S1 — Fungal taxa identified to genus or species level in the wood of grapevine rooted cuttings, at relative abundances (RA) inferior to 0.1 % or 0.01% of the total. [file Table_1.docx]

**Supplementary materials**

**Table S1**. Fungal taxa identified to genus or species level in the wood of grapevine rooted cuttings, at relative abundances (RA) inferior to 0.1 % or 0.01% of the total.

| **RA < 0.1 %** |
| --- |
| *Buckleyzyma* sp. |
| *Cenococcum geophilum* |
| *Colletotrichum gloeosporioides* |
| *Cryptococcus victoriae* |
| *Cyberlindnera jadinii* |
| *Cystofilobasidium infirmominiatum* |
| *Filobasidium magnum* |
| *Hannaella oryzae* |
| *Meira nashicola* |
| *Naganishia albidosimilis* |
| *Neophysalospora eucalypti* |
| *Peniophora piceae* |
| *Phyllosticta* sp. |
| *Pseudozyma* sp. |
| *Rhodotorula mucilaginosa* |
| *Rhodotorula nothofagi* |
| *Saccharomyces cerevisiae* |
| *Sporobolomyces johnsonii* |
| *Stemphylium* sp. |
| **RA < 0.01 %** |
| *Ceratobasidium* sp. |
| *Cladosporium fusiforme* |
| *Coriolopsis gallica* |
| *Cryptococcus heimaeyensis* |
| *Debaryomyces mycophilus* |
| *Debaryomyces prosopidis* |
| *Diaporthe phaseolorum* |
| *Dioszegia* sp. |
| *Erythrobasidium hasegawianum* |
| *Hyphodermella rosae* |
| *Itersonilia perplexans* |
| *Lecanicillium lecanii* |
| *Leucosporidium* sp. |
| *Phaeotheca triangularis* |
| *Pseudotaeniolina globosa* |
| *Tilletiopsis pallescens* |
| *Tubaria furfuracea* |
