# Supplementary material for: Fungicides and the Grapevine Wood Mycobiome: A Case Study on Tracheomycotic Ascomycete Phaeomoniella chlamydospora Reveals Potential for Two Novel Control Strategies
Source: Front Plant Sci. 2019 Oct 31;10:1405. doi: 10.3389/fpls.2019.01405 (PMC6836639; doi:10.3389/fpls.2019.01405)

Figure S1. Rarefaction curves of alpha-diversity indexes; each line correspond to one sample in a range from 0 to 18000 reads. Each box plot represent the distribution of the selected alpha diversity metric for each sample at each 2000 reads of sampling depth. The lower and upper whiskers of the box plot correspond to the 9th and 91st percentiles of the distribution, while the lower and upper extents of the box correspond to the 25th and 75th percentiles of the distribution (respectively). The horizontal bar through the middle of the box is the median of the distribution (i.e., the 50th percentile). Outlier points of these distributions are not shown. The lines connect all the medians from the different. A) Rarefaction curves based on richness, calculated on the number of amplicon sequence variants (ASVs). B) Rarefaction curves based on Shannon index.

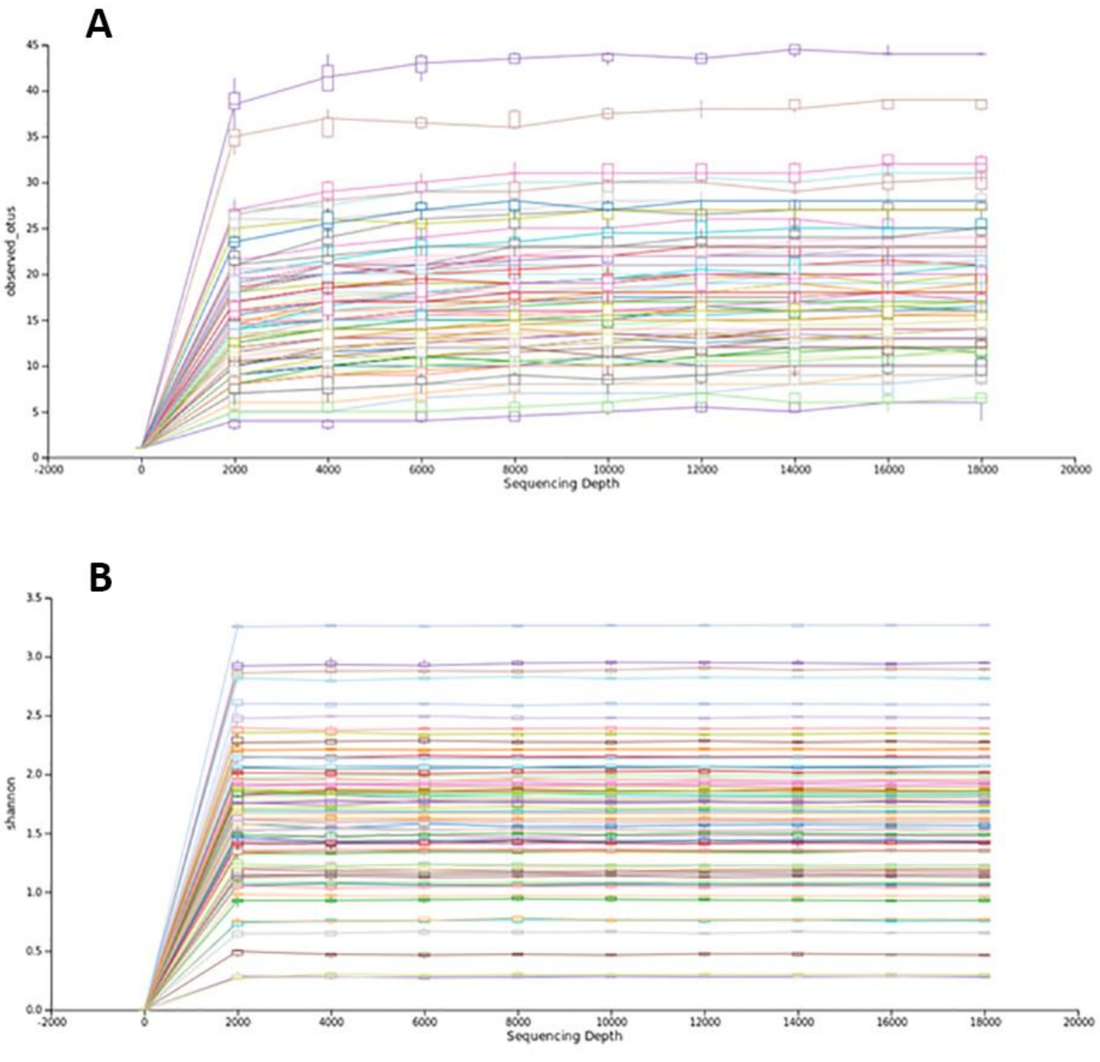

Supplement: Figure S1 — Rarefaction curves of alpha-diversity indexes; each line correspond to one sample in a range from 0 to 18000 reads. Each box plot represent the distribution of the selected alpha diversity metric for each sample at each 2000 reads of sampling depth. The lower and upper whiskers of the box plot correspond to the 9th and 91st percentiles of the distribution, while the lower and upper extents of the box correspond to the 25th and 75th percentiles of the distribution (respectively). The horizontal bar through the middle of the box is the median of the distribution (i.e., the 50th percentile). Outlier points of these distributions are not shown. The lines connect all the medians from the different. A) Rarefaction curves based on richness, calculated on the number of amplicon sequence variants (ASVs). B) Rarefaction curves based on Shannon index. [file Image_1.pdf]
